# Utilization of Partial Cytoreductive Nephrectomy in Patients with Metastatic Renal Cell Carcinoma

**DOI:** 10.3390/jcm13195767

**Published:** 2024-09-27

**Authors:** Nicholas Hauser, Julian Giakas, Hunter Robinson, Facundo Davaro, Zachary Hamilton

**Affiliations:** 1 Saint Louis University School of Medicine, 1402 S Grand Blvd, St. Louis, MO 63104, USA; nicholas.hauser@health.slu.edu (N.H.); julian.giakas@health.slu.edu (J.G.); 2 Geisinger Medical Center, 100 N Academy Ave, Danville, PA 17822, USA; hunter.robinson@health.slu.edu; 3 H. Lee Moffitt Cancer Center & Research Institute, 1600 SW Archer Rd, Gainsville, FL 32608, USA; davarof@ccf.org; 4 Division of Urology, SSM Health Saint Louis University Hospital, 1225 S Grand Blvd, St. Louis, MO 63104, USA

**Keywords:** partial cytoreductive nephrectomy, metastatic renal cell carcinoma, perioperative outcomes, cytoreductive nephrectomy

## Abstract

Objectives: Cytoreductive nephrectomy for metastatic renal cell carcinoma (mRCC) is a standard of care. Partial nephrectomy (PN) in the setting of metastatic disease is an uncommon occurrence, and we aimed to characterize its utilization in a modern cohort. Methods: The National Cancer Database was reviewed for patients with mRCC from 2010 to 2017. Patients with cTanyNanyM1 who underwent cytoreductive surgery in the form of PN or radical nephrectomy (RN) were compiled. Our primary outcome was survival outcome for patients who underwent PN compared to RN. Secondary outcomes included 30-day readmission, length of stay, and survival outcomes. Results obtained: A total of 13,896 patients with mRCC who underwent cytoreductive surgery were identified. In total, 13,242 underwent RN and 654 underwent PN. The RN population was more likely to have cN positive disease, while the PN population was more likely to have cT1 disease. Length of stay, readmission and 30-day mortality were not significantly different between PN and RN, but overall mortality and 90-day mortality favored PN (*p* < 0.001). Cox regression for death showed PN with improved overall survival (HR 0.782, *p* < 0.001). Logistic regression for predictors of cytoreductive PN revealed cT1 and cN0 as significant factors. Overall survival, as seen on KM analysis, identified that PN exhibited improved 2-year (67.1% vs. 52.0%) and 5-year (40.7% vs. 29.2%) overall survival relative to RN (*p* < 0.001). Conclusions: PN is an infrequent treatment with mRCC and its utilization is stable from 2010 to 2017. Overall survival is significantly better for those undergoing PN, likely due to their favorable oncologic disease characteristics.

## 1. Introduction

Renal cell carcinoma (RCC) currently accounts for around 5% of all new cancer diagnoses in men and 3% of all new cancer diagnoses in women worldwide. It also ranks in the top 10 for the most common neoplasms in the United States [1]. This number has been increasing in recent years, as RCC is being found incidentally through unrelated abdominal imaging. While most tumors are found in their early stages of development and are still localized to the kidney, up to 17% of new diagnoses are being made after the cancer has already spread to distant sites throughout the body. The World Health Organization has stated that there are over 140,000 RCC-related deaths annually worldwide and lists it as the 13th most common cause of mortality from cancer [2]. The time and stage of diagnosis play a very significant role in mortality as the 2- and 5-year overall survival rates are 29.3% and 15.5%, respectively, for patients who are diagnosed with distant metastases compared to 96.7% and 93.1% for those who are found with only localized tumors [3]. In patients diagnosed with metastatic RCC (mRCC), treatment may include cytoreductive nephrectomy. Patients who are deemed good candidates for cytoreductive nephrectomy may undergo surgery and systemic therapy before or after the procedure to achieve the best possible survival outcome [4].

Targeted immunotherapies such as tyrosine kinase inhibitors have become a mainstay of treatment for (mRCC), according to guideline statements [5,6]; however, cytoreductive surgery is still an important component of treatment in advanced stage disease [7]. The decision for cytoreduction with stage IV RCC depends on an array of variables including prognostic risk category, comorbidities of the patient, performance status, histology, and patient symptoms [5,8].

Historically, the cytoreductive therapy of choice in the appropriate risk patient has been radical nephrectomy (RN) [9]; however, for cytoreduction, partial nephrectomy (PN) has been utilized in rare circumstances. Current guidelines do not recommend or discuss PN in patients with mRCC [10]. Although overall rates are still low, previous studies have demonstrated increasing rates in cytoreductive PN (CPN) [11]. The role of CPN in the literature remains mixed, with some studies being historical or limited by sample size, and there is a lack of prospective randomized controlled trials. Nonetheless, previous studies have shown that CPN produces similar oncological benefits and acceptable surgical morbidity when compared to cytoreductive RN (CRN) in patients with favorable tumor conditions locally but with distant metastasis [10,12,13,14].

Given the change in recent practice trends and uncertainty regarding the possible effect of CPN on survival of patients with mRCC, it is important to determine the overall survival (OS) outcomes in patients undergoing CPN compared to CRN for the treatment of mRCC, utilizing a large national database. For this study, it was hypothesized that the patients who underwent CPN will have comparable postoperative outcomes and improved OS, as compared to CRN.

## 2. Methods

The National Cancer Database (NCDB) was reviewed for patients with RCC from 2010 to 2017. The NCDB is a joint project of the Commission on Cancer of the American College of Surgeons and the American Cancer Society. The NCDB is a United States cancer outcomes dataset that includes input from over 1500 Commission on Cancer-accredited centers. These data include all cancer patients treated at participating Commission on Cancer-accredited institutions and are estimated to capture over 70% of cancer cases in the United States. Standardized coding definitions are utilized, and the data are freely available to participating institutions after applications for projects are accepted by the NCDB. The data used in the study are derived from a de-identified NCDB file. The American College of Surgeons and the Commission on Cancer have not verified and are not responsible for the analytic or statistical methodology employed, or the conclusions drawn from these data by the investigator. The de-identified information from this database was constructed in a file that included the patient population of interest. Due to the de-identified nature of this retrospective dataset, the current study was exempt from local IRB approval.

The NCDB was queried for patients with cTanyNanyM1 from the renal database, including histology codes for ‘clear cell’, ‘papillary’, ‘chromophobe’, or ‘other’, between 2010 and 2017, who underwent cytoreductive surgery with accurate clinical and pathologic staging data. Patients were categorized based on the clinical tumor stage of disease at time of operation (T1–T4). Charlson Comorbidity Index (CCI) scores (0–3+) as well as patient demographic variables including age, race, sex, insurance coverage, income status, and type of treatment facility (high or low volume) were collected. Deyo modification of CCI was used to divide patients into three groups with CCI scores of 0, 1, and ≥2. In the NCDB database, the calculation of the Charlson–Deyo score excludes any comorbidity code identified as malignant neoplasms from the total score, since all patients have a diagnosis of cancer. Facilities with >500 new cancer diagnoses per year were categorized as high volume, as according to the NCDB dataset. All patients underwent cytoreductive surgery in the form of PN or RN. The utilization of CPN, considering both open and minimally invasive (MIS) approaches, was analyzed. Disease outcomes included all-cause mortality, 30-day mortality, and 90-day mortality. The outcomes were stratified by the type of cytoreductive therapy (CRN vs. CPN).

The primary outcome is overall survival for patients receiving CPN or CRN. The secondary outcomes include length of hospital stay and readmission to the hospital. Multivariable analysis with an increased number of potential confounders was performed for surgical approach and outcomes with Cox regression and logistic regression. Cox regression assessed survival outcomes for all cytoreductive nephrectomies performed due to mRCC with CPN being a subcategory within that dataset, and logistical regression assessed the independent variables affecting a patient’s likelihood of receiving CPN compared to CRN. Normally distributed continuous variables were reported as the mean ± standard deviation (SD) and compared with two-tailed t-tests, analysis of variance (ANOVA). Categorical variables were compared with Chi-square or the Kruskal–Wallis test. Logistic regression, Cox regression, and Kaplan–Meier survival analysis were performed. All statistical analysis was conducted using SPSS v27, and a *p*-value < 0.05 was considered significant.

## 3. Results

Table 1 shows the breakdown of the patient demographics, clinical tumor characteristics, and comorbidity indexes of all the mRCC cases that underwent any type of cytoreductive therapy and separates them according to partial and radical nephrectomy operations. The majority of the cohort underwent a definitive CRN with <5% of the entire cohort receiving CPN.

The mean ages of these patients were almost identical at just over 60 years old and did not differ by surgical strategy. In total, 88.1% of all mRCC patients identified as white. A total of 4.78% of white patients received CPN compared to 4.16% of patients who identified as other races. Men accounted for 70.0% of all recorded cases of mRCC. A total of 91.8% of all patients diagnosed with mRCC had CCI scores of 0 or 1. The presence of significant comorbidities did not differ by surgical strategy. Income level did not differ between cohorts (*p* = 0.144). Only 2.87% of patients who were uninsured underwent CPN.

The clinical stage was the greatest indicator of which type of procedure each patient underwent. In total, 14.72% of mRCC patients who were diagnosed at the cT1 stage received CPN compared to 2.25% in the cT2 group, 1.70% in the cT3 group, and 2.47% of those in the cT4 group (*p* < 0.001). The cT1 subset made up 53.7% of all CPN despite only accounting for 17.2% of all cases involving cytoreductive surgery. Furthermore, 35.9% of all CPNs were performed using minimally invasive techniques compared to 31.5% in CRN cases, and the type of treatment facility did not have an impact on surgical strategy.

Table 2 describes the surgical outcomes of each procedure type and the pathology results following definitive surgical extirpation. CPN patients had a significantly decreased length of post-operative stay and lower rates of readmission. Additionally, the 30- and 90-day mortality rates were lower in CPN patients (1.5% and 6.0% for CPN vs. 2.7% and 10.0% for CRN, *p* < 0.001).

CRN had a 63.6% rate of high-grade tumor, compared to 49.4% for CPN. Clear cell tumors were the most common type of mRCC, accounting for 60.7% of all specimens collected. Chromophobe and papillary were less common and had a higher correlation with CPN than clear cell tumors. In total, 8.28% of all chromophobe tumors and 8.42% of all papillary tumors resulted in CPN compared to 4.45% of cases involving clear cell tumors.

Table 3 shows a Cox regression for all-cause mortality comparing age, comorbidities, clinical stage, grade, and procedure type. Advanced age was a significant predictor of death with a hazard ratio (HR) of 1.010 (*p* < 0.001). Patients with a CCI score of 3+ had a HR of 1.207 (*p* < 0.001). The clinical stages of cT3 and cT4 also had significant correlations (*p* < 0.001) with HRs of 1.143 and 1.397, respectively. Metastasis to distant lymph nodes resulted in an increased outcome of death with an HR of 1.484 (*p* < 0.001). Biopsy specimens with positive margins had an HR of 1.372 (*p* < 0.001). Lastly, histological examination of the excised tumors that were deemed high grade had a statistically significant positive correlation with death with an HR of 1.251 (*p* < 0.001). The only variable with a decreased risk of death was a patient receiving CPN compared to CRN (HR 0.782, *p* < 0.001).

Table 4 is a logistic regression identifying independent risk factors for receipt of CPN compared to CRN. Once again, the variables of patient demographics, clinical tumor characteristics, and comorbidity indexes for all patients who underwent CPN were included. Statistical significance was found with cT1 masses having an OR of 6.183 (*p* < 0.001), while clinically node positive disease predicted a decreased odds of CPN with an OR of 0.629 (*p* < 0.001).

**Figure 1:**
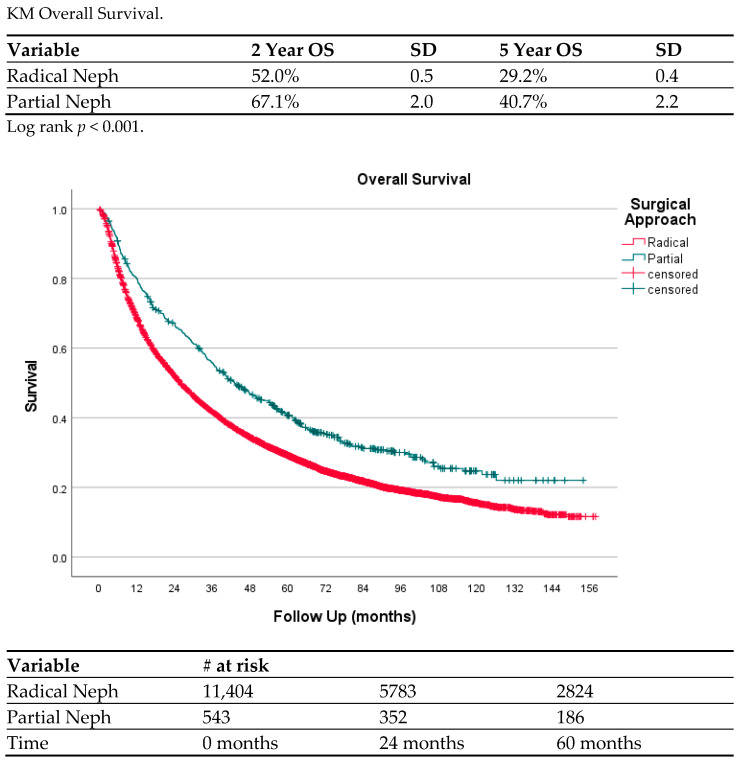
Overall survival between radical and partial cytoreductive nephrectomy.

Figure 1 is a Kaplan–Meyer curve comparing OS rates for patients who underwent CPN vs. CRN. CPN had a greater OS rate at all time points, including 67.1% at 2 years compared to 52.0% for CRN and 40.7% compared to 29.2% at 5 years (*p* < 0.001).

Table 5 shows the proportion of CPN and CRN from 2010 to 2017. There has been an increase in the total number of cytoreductive procedures; however, the percentage of CPN has remained relatively unchanged at around 4.7% to 4.8% during the study period.

Table 6 compares the surgical approach over time for CPN procedures from 2010 to 2017. The percentage of operations using a MIS approach increased substantially over that period going from just 16.0% in 2010 to 42.2% in 2017 (R = 0.659, R^2^ = 0.434, *p* < 0.001).

## 4. Discussion

This study was an analysis of cytoreductive nephrectomy procedures from 2010 to 2017 using the NCDB. Perioperative outcomes and OS rates for patients who underwent CPN were comparable or better to those who underwent CRN, which aligns with the prior literature [8,13,14,15,16,17,18]. These findings may have several implications for future guidelines for the treatment of mRCC.

This study found that CPN in patients with mRCC made up 4.71% of all cytoreductive procedures performed. The decision to perform CPN in the setting of metastatic disease appears driven by cT stage, which likely represents tumor size and complexity. Patients who were diagnosed in the cT1 stage without significant lymph node spread were much more likely to receive CPN than those diagnosed with higher stage disease. In the present study, patients with cT1 disease received CPN 14.72% of the time, and patients who were cN positive were over 1.5 times less likely to receive CPN than their cN negative counterparts. Importantly, nodal status at the time of CPN has been found to impact OS. Chen et al. (2020) found that the risk of death in N1 patients was 5.48 times that of N0 patients, suggesting that higher stage and N1 status have worse outcomes f [14]. Nevertheless, subgroup analysis demonstrated the survival benefit of PN over RN in patients with isolated distant metastasis.

Morphometric data regarding renal masses, such as the RENAL nephrometry score, are not available in this dataset [19]. Furthermore, baseline renal function is not available within the NCDB. The previous literature has suggested that more locally advanced tumors are at higher risk of renal functional decline and mortality, so efforts should be made to preserve renal function when possible [20]. Patient selection is therefore pivotal, as CPN may be beneficial for few cases of mRCC with cT1 primary tumor and oligometastatic disease. This analysis provides support for the decision that CPN can be feasible with acceptable perioperative outcomes in the setting of cT1 tumors.

The data suggest a possible benefit in surgical outcomes and OS for patients who underwent a CPN compared to CRN. Furthermore, the 90-day post procedure mortality rate was 10.0% in CRN patients compared to only 6.0% in CPN patients. This difference in OS persisted at all time points measured up to 5 years postoperatively. These findings qualify as statistically significant differences, showing that CPN are correlated with better survival outcomes. Additionally, surrogate metrics such as the length of postoperative hospital stay and readmission rates were equivalent between CPN and CRN, which suggests that the decision to perform PN in cT1 masses is feasible without compromising patient safety. Similarly, Mazzone et al. (2018) found no significant difference in complication rates (OR: 1.00, *p* = 0.9) and in-hospital mortality (OR: 1.2, *p* = 0.8) between CRN and CPN [18]. However, considering that CPN appeared to be used primarily in situations where the disease had not progressed to the entirety of the kidney, these differences in OS and perioperative outcomes could also be attributed to disease stage rather than surgical strategy.

These differences may also be confounded by a selection bias in favor of CPN patients with smaller primary renal tumors. For example, in a previous study on the utilization and impact of nephron-sparing surgery on survival in those with metastatic disease, only 1% of patients with a primary tumor size ≥ 7 cm were found to undergo CPN [17]. Various studies demonstrate significant differences in primary tumor size in patients with mRCC undergoing treatment by either CRN or CPN. Tian et al. (2023) found that median tumor size was larger in patients undergoing CRN when compared to CPN (7.50 cm vs. 4.95 cm, *p* < 0.001, respectively) [8]. Similarly, another study found mean tumor size was smaller in patients who underwent CPN compared to CRN (5.1 cm vs. 9.3 cm, *p* < 0.001, respectively) [16]. These studies suggest the non-inferior or improved survival benefit of CPN compared to CRN despite the differences in primary tumor size in matched analysis.

Improved technology that has recently begun to be implemented in CPN procedures such as the use of intraoperative ultrasound (IOUS) may lead to an increase in overall survival as negative margins on tumor resection were more likely to be attained. Mihai et al. (2024) found that in procedures utilizing IOUS there was a higher rate of negative margins along with significantly reduced blood loss, a shorter median operative duration, and diminished ischemia time (*p* = 0.001) [21]. These results suggest that the new advancements could have a large impact on overall survival for patients undergoing CPN with the use of IOUS compared to those receiving CRN.

The RN group had a higher proportion of clear cell RCC (ccRCC) (60.9% vs. 57.5%, *p* < 0.001). Interestingly, pathologic-type non-ccRCC, despite being less common, was more commonly associated with CPN. Previous studies have shown ccRCC is more common in CRN than CPN (73.5% vs. 63.5%, *p* = 0.052), and that non-ccRCC is a risk factor for OS and cancer-specific survival (CSS) (OS: HR = 1.69, *p* = 0.002; CSS: HR = 1.51, *p* = 0.021) [8]. Given the survivability benefit of CPN observed in the present study and improved outcomes for ccRCC compared to non-ccRCC seen in the literature, pathologic-type ccRCC may therefore be a positive predictor of OS and CSS in patients with mRCC who undergo PN.

While the direct clinical stage of the disease played a significant role in a patient’s likelihood of undergoing CPN, overall health did not seem to be greatly correlated. Patients with mRCC who had a CCI score of 3+, which designates them as someone with many comorbidities, still had a 4.49% chance of receiving a CPN; this was relatively identical to the 4.59% of patients with a CCI score of 0 who underwent the same procedure. However, these patients with a 3+ CCI score still had a much lower OS rate with an HR of 1.207 compared to the patients with fewer comorbidities. Comorbidity has been shown to decrease survival in patients with RCC, irrespective of age, despite increasing survival over time; moreover, mortality rates were higher in patients with CCI scores of 1–2 (HR 1.15, 95%CI 1.06–1.24) and 3 (HR 1.56, 95%CI 1.40–1.73) compared to patients with no registered comorbidity [22]. Furthermore, although increasing comorbidities produces worse outcomes for patients with RCC, the current study suggests the decision to perform CPN in patients with mRCC is driven by mass size and technical feasibility, as opposed to patient baseline health status.

As expected, the total number of cytoreductive procedures increased steadily from 2010 (1561) to 2017 (1895), in response to the increased incidence rate of mRCC in the population and advancements in diagnostic tools. Interestingly, the overall proportion of CPN compared to all cytoreductive kidney procedures did not change substantially from 2010 to 2017 (4.8% and 4.7%, respectively). This is in contrast to a separate study by Lenis et al. (2018), where authors used the NCDB to evaluate trends in CPN during an earlier time frame and found increased rates of CPN from 1.8% to 4.3% between 2006 and 2013 [11]. The same study found that patients with mRCC presenting in academic/research institutions were more likely to receive CPN during the same period (OR = 1.44, *p* = 0.004). Moreover, this analysis found a substantial increase in the utilization of minimally invasive techniques to perform CPN from just 16.0% of procedures in 2010 to 42.2% in 2017. This may correspond to the increase in cytoreductive techniques being performed at academic/research institutions or also to the recent advancements in minimally invasive management of localized and locally advanced RCC [23]. Future research may focus on the use of minimally invasive surgery as a driver of the CPN approach to metastatic disease.

From a financial perspective, the cost of treating RCC was estimated to be USD 2.1 billion as of 2015 with a significant portion of that expense coming from hospital stays. Additionally, patients diagnosed with mRCC had seven times greater cost on average compared to patients diagnosed in the earlier stages of the disease [6]. Because of this, the prolonged hospital treatment of patients with mRCC can cause serious strain on a hospital’s personnel and financial resources. The average length of stay for all cytoreductive procedures was 5.0 ± 5.9 days, and it was slightly shorter for patients who underwent a partial cytoreductive nephrectomy at 4.6 ± 6.4 days (0.06). Although not statistically significant, a difference in the length of stay between radial and partial cytoreductive nephrectomy may have real impacts on the financial burdens of patients and hospital systems. Being able to perform a procedure that has a significantly shorter average hospital stay for patients is crucial to avoiding unnecessary expenses for the patient and hospital, as well as allowing for the best treatment in the most efficient manner possible.

This study was limited by the retrospective nature of the data and the inherent selection bias. Many important variables are not captured by the NCDB, including morphometric data, renal function, overall metastatic burden, patient symptoms, and individual decision choices between the CPN and CRN approaches. While the study did attempt to show how there are many underlying variables that have a significant impact on which surgical strategy patients received, it was unable to further stratify these datapoints between the two groups, leaving room for confounders in the outcomes and therefore further studies with more homogenous control groups could be beneficial in the future. Also, this dataset only included patients from 2010 to 2017 and therefore may not be perfectly representative in a modern population that has access to targeted immunotherapies, such as tyrosine kinase inhibitors, that have become more available in recent years and may have led to improved survival outcomes in patients with mRCC undergoing CN [24,25]. However, this analysis represents the most accurate and thorough national description of this procedure at the time of publication. Importantly, these data were collected from patients before the release of the CARMENA trial, which has sparked significant debate on the timing of cytoreductive surgery and ideal patient selection [26]. Despite these limitations, this study is hypothesis generating and shows the feasibility of CPN for a select group of metastatic patients. This provides correlational analysis for the implantation of different types of cytoreductive therapies on a variety of perioperative outcomes and OS.

## 5. Conclusions

Review of a national dataset from 2010 to 2017 shows that approximately 5% of metastatic kidney cancer patients that receive cytoreductive surgery undergo CPN. The proportion of this surgical approach has not changed over time; however, the use of MIS for CPN has increased, and CPN is significantly correlated with cT1 tumor stage. Survival outcomes appear improved for patients receiving CPN, but this is likely due to patient selection. Further research is necessary to determine which patients are best suited for this approach and to consider guideline statements.

## Figures and Tables

**Table 1:** Patient demographics, clinical tumor characteristics, and survival.

Variable	All Cytoreductive (n = 13,896)	Radical Neph (n = 13,242)	Partial Neph (n = 654)	*p*-Value
Mean Age	61.4 ± 10.8	61.4 ± 10.7	61.6 ± 10.8	0.585
Race				0.530
White	12,236 (88.1%)	11,651 (88.0%)	585 (89.4%)	
Black	1009 (7.3%)	967 (7.3%)	42 (6.4%)	
Other	651 (4.7%)	624 (4.7%)	27 (4.1%)	
Male	9723 (70.0%)	9258 (69.9%)	46 (71.1%)	0.540
Charlson				0.549
0	9974 (71.8%)	9516 (71.9%)	458 (70.0%)	
1	2782 (20.0%)	2646 (20.0%)	136 (20.8%)	
2	739 (5.3%)	697 (5.3%)	42 (6.4%)	
3+	401 (2.9%)	383 (2.9%)	18 (2.8%)	
Income Status				0.144
<USD 38,000	1943 (14.0%)	1868 (14.1%)	75 (11.5%)	
USD 38,000–47,999	2856 (20.6%)	2720 (20.5%)	136 (20.8%)	
USD 48,000–62,999	3441 (24.8%)	3288 (24.8%)	153 (23.4%)	
USD 63,000+	3811 (27.4%)	3623 (27.4%)	188 (28.7%)	
Unknown	1845 (13.3%)	1743 (13.2%)	102 (15.6%)	
Uninsured	522 (3.8%)	507 (3.8%)	15 (2.3%)	0.056
cT Stage				<0.001
cT1	2385 (17.2%)	2034 (15.4%)	351 (53.7%)	
cT2	4037 (29.1%)	3946 (29.8%)	91 (13.9%)	
cT3	4636 (33.4%)	4557 (34.4%)	79 (12.1%)	
cT4	890 (6.4%)	868 (6.6%)	22 (3.4%)	
cTx	1948 (14.0%)	1837 (13.9%)	111 (17.0%)	
cN positive	3391 (24.4%)	3304 (25.0%)	87 (13.3%)	<0.001
Neoadjuvant	939 (6.8%)	895 (6.8%)	44 (6.7%)	0.992
Minimally Invasive	4372 (31.5%)	4137 (31.2%)	235 (35.9%)	0.012
High Volume Center	10,459 (75.3%)	9967 (75.3%)	492 (75.2%)	1.000

**Table 2:** Surgical outcomes and pathology.

Variable	All Cytoreductive (n = 13,896)	Radical Neph (n = 13,242)	Partial Neph (n = 654)	*p*-Value
Length of Stay	5.0 ± 5.9	5.0 ± 5.9	4.6 ± 6.4	0.06
Readmission	724 (5.2%)	699 (5.3%)	25 (3.8%)	0.110
Positive Margin	2688 (19.3%)	2587 (19.5%)	101 (15.4%)	0.010
High Grade	8745 (62.9%)	8422 (63.6%)	323 (49.4%)	<0.001
Length of Follow Up (months)	37.1 ± 34.3	36.6 ± 34.0	48.0 ± 38.0	<0.001
Mortality	10,637 (76.5%)	10,206 (77.1%)	431 (65.9%)	<0.001
30-day	368 (2.6%)	358 (2.7%)	10 (1.5%)	0.087
90-day	1364 (9.8%)	1325 (10.0%)	39 (6.0%)	<0.001
Pathology				<0.001
Clear cell	8441 (60.7%)	8065 (60.9%)	376 (57.5%)	
Chromophobe	169 (1.2%)	155 (1.2%)	14 (2.1%)	
Papillary	653 (4.7%)	598 (4.5%)	55 (8.4%)	
Other	4633 (33.3%)	4424 (33.4%)	209 (32.0%)	

**Table 3:** Cox regression for death.

Variable	HR	95%CI Low	95%CI High	*p*-Value
Age	1.010	1.008	1.012	<0.001
Charlson Score (0 ref)				<0.001
1	1.016	0.968	1.066	0.517
2	1.127	1.036	1.226	0.005
3+	1.207	1.079	1.351	0.001
Clinical Stage (cT1 ref)				<0.001
cT2	1.062	0.999	1.129	0.053
cT3	1.143	1.076	1.213	<0.001
cT4	1.397	1.277	1.529	<0.001
cTx	1.252	1.167	1.343	<0.001
cN Positive	1.484	1.420	1.552	<0.001
Partial Nephrectomy	0.782	0.708	0.863	<0.001
Positive Margin	1.372	1.307	1.441	<0.001
High Grade	1.251	1.202	1.303	<0.001

**Table 4:** Logistic regression for partial (exclude cTx).

Variable	OR	95%CI Low	95%CI High	*p*-Value
Mean Age	0.993	0.984	1.001	0.090
Race (white ref)				
Black	0.906	0.627	1.310	0.600
Other	1.020	0.655	1.589	0.929
Male	1.055	0.866	1.285	0.596
Charlson (0 ref)				
1	1.015	0.813	1.267	0.894
2	1.160	0.799	1.683	0.436
3+	0.755	0.438	1.301	0.311
Income Status (USD 63,000+ ref)				
USD 38,000>	1.074	0.808	1.429	0.622
USD 38,000–47,999	0.798	0.584	1.091	0.158
USD 48,000–62,999	0.900	0.696	1.164	0.422
Unknown	0.869	0.679	1.114	0.268
Uninsured	0.631	0.339	1.176	0.147
cT Stage (cT4 ref)				
cT1	6.183	3.972	9.627	<0.001
cT2	0.830	0.517	1.334	0.442
cT3	0.648	0.401	1.047	0.077
cN Positive	0.629	0.490	0.807	<0.001
High Volume Center	1.126	0.912	1.392	0.270

**Table 5:** Surgical approach over time.

Variable	Partial Neph (n = 654)	Radical Neph (n = 13,242)	All Cytoreductive (n = 13,896)
2010	75	1486	1561
	4.8%	95.2%	100.0%
2011	62	1490	1552
	4.0%	96.0%	100.0%
2012	79	1515	1594
	5.0%	95.0%	100.0%
2013	89	1641	1730
	5.1%	94.9%	100.0%
2014	91	1757	1848
	4.9%	95.1%	100.0%
2015	83	1761	1844
	4.5%	95.5%	100.0%
2016	85	1787	1872
	4.5%	95.5%	100.0%
2017	90	1805	1895
	4.7%	95.3%	100.0%
All Years	654	13,242	13,896
	4.7%	95.3%	100.0%

**Table 6:** Minimally invasive surgical approach over time.

Variable	Open Partial (n = 311)	Min Inv Partial (n = 180)	All Partial Nephrectomy (n = 654)
2010	63	12	75
	84.0%	16.0%	100.0%
2011	46	16	62
	74.2%	25.8%	100.0%
2012	62	17	79
	78.5%	21.5%	100.0%
2013	62	27	89
	69.7%	30.3%	100.0%
2014	55	36	91
	60.4%	39.6%	100.0%
2015	36	47	83
	43.4%	56.6%	100.0%
2016	43	42	85
	50.6%	49.4%	100.0%
2017	52	38	90
	57.8%	42.2%	100.0%
All Years	419	235	654
	64.1%	35.9%	100.0%
